# Development and validation of forensically useful growth models for Central European population of *Creophilus maxillosus* L. (Coleoptera: Staphylinidae)

**DOI:** 10.1007/s00414-020-02275-3

**Published:** 2020-04-08

**Authors:** Katarzyna Frątczak-Łagiewska, Andrzej Grzywacz, Szymon Matuszewski

**Affiliations:** 1 Laboratory of Criminalistics, Adam Mickiewicz University, Św. Marcin 90, 61-809 Poznań, Poland; 2 Centre for Advanced Technologies, Adam Mickiewicz University, Uniwersytetu Poznańskiego 10, 61-614 Poznań, Poland; 3 Department of Animal Taxonomy and Ecology, Adam Mickiewicz University, Uniwersytetu Poznańskiego 6, 61-614 Poznań, Poland; 4 Department of Ecology and Biogeography, Faculty of Biological and Veterinary Sciences, Nicolaus Copernicus University, Lwowska 1, 87-100 Toruń, Poland

**Keywords:** Forensic entomology, Developmental models, *Creophilus maxillosus*, Staphylinidae, Minimum postmortem interval, Validation study

## Abstract

The hairy rove beetle, *Creophilus maxillosus* (Linnaeus) (Staphylinidae), is recognized for its use in forensic entomology. However, insufficient developmental data exist for the Central European population of this species. Accordingly, we studied the development of *C. maxillosus* at ten constant temperatures (10–32.5 °C). Based on these results, linear and nonlinear developmental models were created and validated*.* We also studied the effect of different homogenous diets (third-instar larvae or puparia of *Calliphora* sp. Robineau-Desvoidy or *Lucilia* sp. Robineau-Desvoidy (Diptera: Calliphoridae) or mix of first- and second-instar larvae of *Necrodes littoralis* (Linnaeus) (Coleoptera: Silphidae)) on the development and mortality of *C. maxillosus.* Average total development times ranged between 122.21 days at 15 °C and 22.18 days at 30 °C. Beetles reached the adult stage in seven out of ten temperatures (15–30 °C). No beetles reached the adult stage when fed with larvae of *N. littoralis*; their development times at first and second larval stage were also significantly longer than in other food conditions*.* When *C. maxillosus* larvae were fed with blowfly larvae, the highest mortality was observed at the pupal stage, as compared when they were fed with blowfly puparia—at the first larval stage*.* While validating thermal summation models, the highest age estimation errors were found for beetles bred at 10 and 12.5 °C (between 21 and 43% for all developmental events). Age estimation errors were on average higher for pupation and eclosion than hatching and first and second ecdyses. While validating the models with specimens fed with different diets, the highest errors were recorded for beetles fed with *N. littoralis* larvae (22% for the first ecdysis and 33% for the second ecdysis) and *Lucilia* sp. puparia (32% for pupation and 22% for eclosion). Implications for *C. maxillosus* use in forensic entomology are discussed.

**Electronic supplementary material:**

The online version of this article (10.1007/s00414-020-02275-3) contains supplementary material, which is available to authorized users.

**Table:** 

**Key Points** • Development of the Central European population of *C. maxillosus* was studied at ten constant temperatures and using different homogenous diets. • Thermal summation models were validated with insects reared at different temperatures and fed with different diets. • Total development times ranged between 122 days at 15 °C and 22 days at 30 °C. Beetles reached the adult stage in seven temperatures (15–30 °C). • The highest age estimation errors were found for beetles bred at 10 and 12.5 °C (21–43%) and for beetles fed with *Necrodes littoralis* larvae (22–33%). • The lowest mortality was observed for beetles fed with *Calliphora* sp. and *Lucilia* sp. larvae. Estimation errors were generally low for beetles fed with blowfly larvae or *Calliphora* sp. puparia.	

## Introduction

Development time of insects depends mostly on temperature [1, 2]. Poikilotherms operate within species-specific temperature ranges associated with their local temperatures. Development beyond these limits may be harmful or even lethal for them [3, 4]. Predicted thermal tolerance for development of insects, the range in temperature between the minimum and the maximum rate of development, is about 20 °C [5]. Thermal development of insects is a powerful tool in forensic entomology. Insect development models may be used to estimate the minimum time that elapsed from death until the body discovery (called min PMI) [2]. This involves estimating the age of the oldest immature insects found on a cadaver based on age indicators such as development stage or larval length [2]. The possibility to use the method depends on the availability of developmental data for the species collected on a crime scene. Observed values of insect age indicators are compared with the reference developmental data. Such data are collected during laboratory experiments where the development of insects is studied under controlled conditions [6]. Results of such experiments are presented using graphical development representations (i.e., isomegalen and isomorphen diagrams) or mathematical development models (i.e., linear or nonlinear development equations) [7].

Developmental data used in forensic entomology are collected using different insect rearing protocols, sampling frequency, sample sizes, killing or preservation techniques etc. As a consequence, the data used in casework may differ in quality with likely detrimental effects on the accuracy or precision of min PMI estimates [8]. The ideal reference data should accurately reflect the development of insects on cadavers under natural conditions. However, developing such perfect reference data in the laboratory is simply impossible. Therefore, all data used by forensic entomologists in casework have limitations resulting from their laboratory origin. Currently, one of the most pressing needs of forensic entomology is to identify factors affecting the quality of reference developmental data and to determine the magnitude of their impact.

Insect development was analyzed and modeled in different ways in forensic entomology [9]. In most cases, linear development models were used, i.e., simple regression model relating developmental rate with rearing temperature [2] or the thermal summation model sensu Ikemoto and Takai [10] (e.g., [11–16]). Although frequently used in insect developmental studies, nonlinear models were developed less frequently in forensic entomology–oriented experiments (but see [9, 17–19]). Although they describe the relationship more accurately than linear models, they are less practically useful as they do not provide thermal constant *K* for the developmental landmarks [17]. The constant *K* for a landmark may be divided into smaller parts, enabling retrospective calculation of the time needed to reach the landmark in changing temperature conditions. Moreover, it may be used to estimate the age of the most problematic pieces of insect evidence, i.e., postfeeding larvae or pupae, through subtracting thermal units which insects collected on a crime scene have accumulated in the laboratory from the constant *K* [20]. The thermal constants may be estimated using different methods and from different data. Accordingly, they may represent the true thermal constants for an insect population with different accuracy. For this reason, it is necessary to validate thermal constants or any other reference developmental data used by forensic entomologists [21]. Some validation studies in forensic entomology revealed low accuracy of the laboratory-produced data when applied to insects reared in naturally occurring temperatures [21]. Although there are different ways to validate reference developmental data [21–25], the minimum standard should be a validation using insect laboratory sample different than the one used for the derivation of the data.

Another group of factors affecting the quality of developmental data is related to laboratory protocols, e.g., methods of larval measurement, conditions of insect colony maintenance, or food quality and quantity [26]. Food quality influences the survival and development of insects in nature [27]. Its effect on the development was demonstrated in several species of necrophagous flies [21, 28–32]. No previous work tested such effects in forensically useful insect predators. Prey quality has a direct impact on growth and development of predatory insects [33, 34]. It is not clear what exactly predatory beetle species (e.g., staphylinids or histerids) eat on carrion. Based on the literature [35, 36], they should prey mostly on larvae and puparia of blowflies. However, their food preferences have not been investigated. Moreover, it is possible that food type affects their development. Consequently, in casework, the accuracy of insect age estimation may be reduced by using developmental data derived from insects fed with non-optimal food type.

*Creophilus maxillosus* (Linnaeus) (Coleoptera: Staphylinidae) is a common predator of fly larvae and puparia associated with carrion [7, 37]. It abundantly visits and breeds in large vertebrate cadavers and is recognized as highly forensically useful [38–41]. Early works analyzed the development of *C. maxillosus* in uncontrolled rearing conditions [42, 43]. Watson-Horzelski [44] investigated the development of *C. maxillosus* at 3 temperatures for the population from Southern United States. Wang et al. [45] provided more robust development models of the species for the population from China. However, distinct populations may respond differently to similar environmental conditions. Thus, to get accurate insect age estimates, calculations should use developmental data for the local insect population [17, 46]. Recently more attention has been paid to the Central European population of *C. maxillosus* [7, 8, 47]. Differences in development time between males and females of *C. maxillosus* were reported, however with no significant influence on the accuracy of age estimates using sex-specific as compared with general thermal summation models [7]. *Creophilus maxillosus* size at emergence was demonstrated to be useful for physiological age prediction and accordingly for the improvement of the age estimate accuracy in forensic entomology [47]. Moreover, it was found that the multiple measurement protocol affects the accuracy of insect age estimates using the resultant reference developmental data [8]. However, none of these studies derived developmental models for all developmental landmarks of Central European *C. maxillosus*.

Consequently, the aim of this study was to create full set of developmental models for the central European population of *C. maxillosus* and to validate the models with insects reared at different temperature conditions and fed with different diets. Additionally, we investigated how different homogenous diets affect development and mortality of *C. maxillosus*.

## Materials and methods

### Insect colony establishment and maintenance

Insects were collected from rabbit carcasses placed in a xerothermic grassland (Biedrusko military range, western Poland: 52 31′ N, 16 55′ E). Carcasses were exposed in spring and summer of 2015 and 2016 every few days to have permanent access to adult beetles. Beetles were collected during 5–7 inspections each year, 10–15 specimens at a time. A colony consisted of 25–30 individuals with a more or less equal proportion of males and females. New beetles sampled in the field and first generations bred in the laboratory were used to permanently renew the colony. Adult beetles were kept in plastic containers (30 × 20 × 20 cm) with 6–7 cm layer of moist soil and access to water. They were fed once a day with a mix of blowfly third-instar larvae and puparia. Containers were kept at room temperature (20–22 °C) and humidity (50–60%) and cleaned once a week to avoid the appearance of mites and mold.

### Methods common for temperature and food type experiments

#### Rearing

Females of *C. maxillosus* lay singular eggs in small clumps of soil which makes them difficult to be found. Eggs in the same age were obtained by placing adult insects from a single colony into 3-l container filled halfway with soil for 4 h. Containers were kept in the dark at a room temperature (20–22 °C). Afterwards, adult beetles were pulled out and containers were placed in insect incubators (ST 1/1 BASIC or +, POL-EKO, Poland) set for the specific temperature. After 70% of the average egg stage duration, containers were inspected for the presence of first-instar larvae at intervals equal to 10% of the average egg stage duration. Freshly hatched first-instar larvae are creamy-white and very active, so it is easy to find them while searching the soil. Only freshly hatched larvae were sampled and transferred to separate cups.

First- and second-instar larvae were kept in 80-ml containers filled with 1.5 cm of soil. Third-instar larvae, immediately after the second ecdysis, were transferred to 120-ml containers with 5–6 cm of soil and were kept there until adults emerged. Containers were placed in insect incubators (ST 1/1 BASIC or +, POL-EKO, Poland). Humidity in incubators was maintained at 60–70% and a photoperiod (h) was set on 12:12 (L:D).

#### Inspections and measurements

All individuals were inspected for developmental landmarks: hatching, first and second ecdysis, pupation, adult emergence. After 60% of the average stage duration, insects were checked every 10% of stage duration. Containers were taken out of the incubator, and beetles were inspected for developmental stage. In each stage, 4–5 inspections were made. After noticing the landmark, the midpoint between current and previous inspection was used as the actual time of the landmark occurrence. Transitions between larval stages were determined based on the creamy-white color of a larva (appearing shortly after ecdysis) and the width of the mesonotum.

A geometrical micrometer was used to measure in vivo larval length. The larva was placed in a 1.5-ml Eppendorf tube, and after it had become immobile and fully erected, its length (from clypeus to the last abdominal segment) was measured with a micrometer. The analytical balance AS 82/220.R2 (Radwag, Poland) was used to weigh larvae and pupae in a 1.5-ml Eppendorf tube.

### Experiment 1: Effect of temperature on development

Development was studied at ten constant temperatures: 10–32.5 °C, in 2.5 °C intervals. Two or three temperatures were studied at the same time. Larvae were fed once a day with third-instar larvae of blowflies punctured to make feeding easier for the first- and second-instar larvae of *C. maxillosus*.

Forty larvae per temperature were used. Insects were randomly allocated to temperatures. All individuals were inspected for developmental landmarks. Twenty individuals were also repeatedly measured and weighted. Containers were placed on two shelves inside the incubator. Container positions were rearranged every few inspections.

Inspection intervals were calculated based on the results of pilot tests at 5 temperatures (12.5, 17.5, 22.5, 27.5, 32.5 °C; 10 insects per temperature). Intervals established at a specific temperature were also used for the lower adjoining temperature (e.g., intervals at 12.5 °C were also used for 10 °C).

Differences in mortality between specimens bred at different temperatures were evaluated using the chi-squared test. Percentage mortality was defined as [(number of dead specimens × 100%)/number of sampled larvae]. Differences in time of development and differences in length or weight between specimens bred at different temperatures were evaluated using one-way analysis of variance. All analyses were conducted using Statistica 13.1 (StatSoft).

Models for particular developmental events were developed using data for randomly selected non-measured beetles (usually 10 per temperature), chosen for each model from the entire insect pool. We derived the linear Ikemoto and Takai model [10] (Eq. 1) and nonlinear models: Analytis [48] (Eq. 2), Brière-2 [49] (Eq. 3), Lactin-2 [50] (Eq. 4), and SSI (Sherpe-Schoolfield-Ikemoto) [51] (Eq. 5).

1 $$ DT=k+{T}_{\mathrm{min}}D $$

where *D* is the duration of development (in hours or days), *T* is the rearing temperature, *T*_min_ is the lower developmental threshold, and *k* is thermal summation constant. It is recommended to calculate these parameters by means of the reduced major axis (RMA) instead of the ordinary least squares (OLS) regression [10, 17]. Model was calculated with the *lmodel2* package [52] in *R*, where the RMA is called a standard major axis (SMA) regression.

2 $$ \frac{1}{D}=a\times {\left(T-{T}_{\mathrm{min}}\right)}^n\times {\left({T}_{\mathrm{max}}-T\right)}^m $$

3 $$ \frac{1}{D}=a\times \left(T-{T}_{\mathrm{min}}\right)\times {\left({T}_{\mathrm{max}}-T\right)}^{\frac{1}{d}} $$

4 $$ \frac{1}{D}={e}^{\left(p\times T\right)}-{e}^{\left(p\times {T}_{\mathrm{max}}-\left(\frac{T_{\mathrm{max}}-T}{\Delta  T}\right)\right)}+\lambda $$

5 $$ \frac{1}{D}=\frac{\ {\rho}_{\upvarphi \kern0.5em }\left(\frac{T}{T_{\varphi }}\right)\times {e}^{\left[\frac{{\Delta  H}_{\mathrm{A}}}{R}\times \left(\left(\frac{1}{T_{\upvarphi}}\right)-\left(\frac{1}{T}\right)\right)\right]}}{1+{e}^{\left[\frac{{\Delta  H}_{\mathrm{L}}}{R}\times \left(\left(\frac{1}{T_{\mathrm{L}}}\right)-\left(\frac{1}{T}\right)\right)\right]}+{e}^{\left[\frac{{\Delta  H}_{\mathrm{H}}}{R}\times \left(\left(\frac{1}{T_{\mathrm{H}}}\right)-\left(\frac{1}{T}\right)\right)\right]}} $$

where 1/*D* is development rate, *T* is the rearing temperature, *T*_min_ is the lower developmental threshold, and *T*_max_ is the upper developmental threshold [53]. In the Analytis model *a*, *n*, and *m* are constants [48, 54]. In the Brière-2 model, *a* and *d* are empirical constants [49, 55]. In the Lactin-2 model, *p* is a constant defining rate of optimum temperature, Δ*T* is the temperature range across which physiological breakdown becomes the overriding influence, and *λ* allows the curve to intercept the *x*-axis allowing the estimation of lower temperature threshold (*T*_min_) [50, 54]. In the SSI model, *ρ*_φ_ is the development rate at the intrinsic optimum temperature *T*_φ_, Δ*H*_A_ is the change in enthalpy of activation of the reaction that is catalyzed by the enzyme, Δ*H*_L_ is the change in enthalpy associated with low temperature inactivation of the enzyme, and Δ*H*_H_ is the change in enthalpy associated with high temperature inactivation of the enzyme, *R* is the gas constant (1.987 cal/deg/mol), *T*_L_ is the temperature at which the enzyme is 1/2 active and 1/2 low temperature inactive, and *T*_H_ is the temperature at which the enzyme is 1/2 active and 1/2 high temperature inactive (both in Kelvin degrees) [51, 53].

Fitting of the nonlinear Analytis, Brière-2, and Lactin-2 models was done using the Levenberg-Marquardt algorithm with the *minpack.lm* package [56] and the SSI model using the *SSI* package [51], both in *R*.

### Experiment 2: Effect of food type on development

For the experiment, we chose five different types of food, i.e., third-instar larvae of *Calliphora* sp. Robineau-Desvoidy and *Lucilia* sp. Robineau-Desvoidy (Diptera: Calliphoridae), puparia of *Calliphora* sp. and *Lucilia* sp., mix of first- and second-instar larvae of *Necrodes littoralis* (Linnaeus) (Coleoptera: Silphidae), as they are present on carrion at the same time as larval stages of *C. maxillosus* [57]. In these food type conditions, we reared 25, 20, 20, 25, and 20 larvae of *C. maxillosus*, respectively. Third-instar larvae of blowflies were purchased from a fishing shop. Genus determinations were made using the identification key by Szpila [58]. In order to get puparia, larvae were bred in the laboratory. First- and second-instar larvae of *N. littoralis* were sampled from our laboratory colony.

Development was studied under constant temperature conditions of 24 °C. Larvae were fed once a day ad libitum. All beetles were inspected for developmental landmarks. Additionally, third-instar larvae were measured and weighed at the beginning of the third larval stage and pupae were weighed at the beginning of the pupal stage.

Differences in mortality between specimens fed with different types of food were evaluated using the chi-squared test. Percentage mortality was defined as [(number of dead specimens × 100%)/number of sampled larvae]. Differences in time of development and differences in length or weight between specimens fed with different types of food were evaluated using one-way analysis of variance. All analyses were conducted using the Statistica 13.1.

### Validation of development models

Models for particular developmental events were validated using different number of specimens originating from different temperature ranges, i.e., for hatching—241 specimens bred at 10–32.5 °C, for first ecdysis—77 specimens bred at 12.5–32.5 °C, for second ecdysis—75 specimens bred at 12.5–32.5 °C, for pupation—46 specimens bred at 17.5–30 °C, and for eclosion—29 beetles bred at 20–27.5 °C. Due to large mortality at extreme temperatures, some of them were poorly represented or not represented in the validation sample. Models were also validated using beetles fed with different food types from our experiment 2, with 104, 102, 100, 82, and 72 beetles respectively for hatching, first ecdysis, second ecdysis, pupation, and eclosion.

The validation included a comparison of the thermal units needed to reach a particular developmental landmark with the thermal constant from the model. For this purpose, we calculated absolute differences between actual and model thermal units and divided them by actual thermal units.

## Results

### Development of *C. maxillosus* across temperatures

**Fig. 1:**
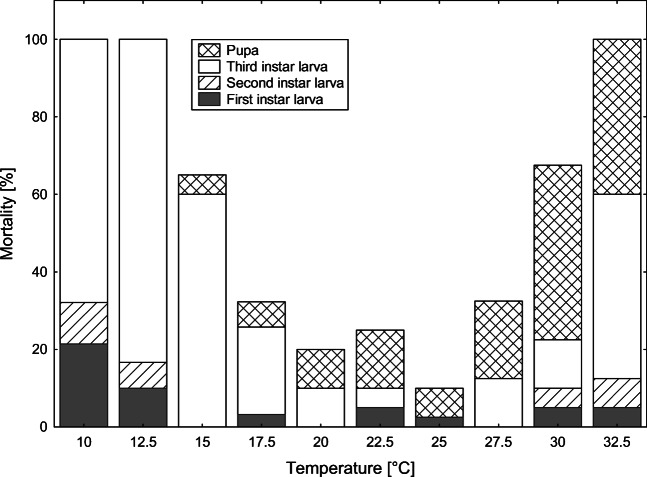
Mortality of *C. maxillosus* developmental stages at different rearing temperatures

Mortality significantly varied across the temperatures (*χ*^2^ = 420.0579, *P* < 0.001; Fig. 1). No beetle reached the adult stage at 10, 12.5, and 32.5 °C. The lowest mortality was at 25 °C (Fig. 1). The highest mortality was recorded for third-instar larvae and pupae (Fig. 1). Mortality of third-instar larvae was the highest at low and high extreme temperatures, whereas pupae revealed an increase in mortality with increase in temperature (Fig. 1).

**Fig. 2:**
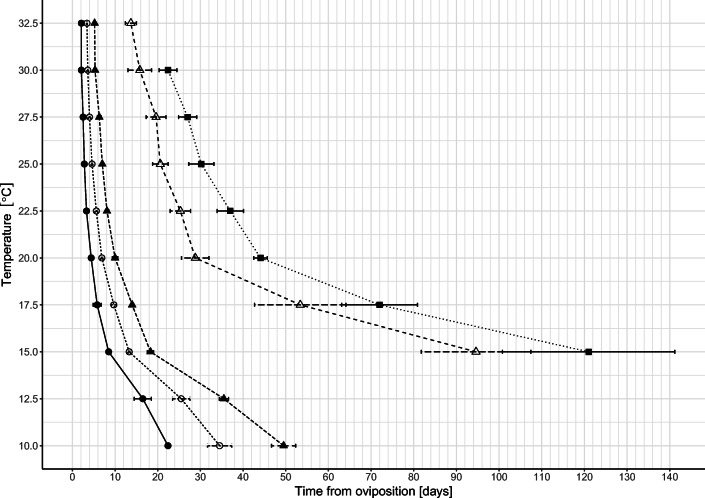
Isomorphen diagram for *C. maxillosus* based on median times to reach particular developmental events at each of the rearing temperature conditions. Horizontal bars represent interquartile ranges. Areas between lines represent developmental stages; symbols represent developmental events. Black circle—hatching. White circle—first ecdysis. Black triangle—second ecdysis. White triangle—pupation. Black square—eclosion

Beetles reached the adult stage in seven out of ten temperatures (15–30 °C) (Table 1, Figs. 1 and 2). Total development time ranged between 122.21 days at 15 °C and 22.18 days at 30 °C (Table 1). At 10 and 12.5 °C, most larvae died in the postfeeding phase (Table 1, Figs. 1 and 2). At 32.5 °C, most larvae pupated but then pupae died (Table 1, Figs. 1 and 2). Fresh pupae reared at 30 and 32.5 °C often failed to shed the third-instar exuvia.

**Table 1:** Time of development (mean (SE; *N*)) of *C. maxillosus* at 10 constant temperatures. Mean for the egg stage duration was calculated on the basis of measured and non-measured individuals. For other stages, mean was calculated using only non-measured individuals

Temperature (°C)	Egg	1st-instar larva	2nd-instar larva	3rd-instar larva	Pupa	Total development
10	22.9 (0.24; 19)	12.9 (0.54; 9)	14.06 (0.27; 8)	-	-	-
12.5	16.58 (0.18; 25)	8.92 (0.14; 13)	9.89 (0.22; 14)	-	-	-
15	8.41 (0.04; 40)	4.99 (0.1; 20)	5.09 (0.12; 20)	67.74 (10.81; 5)	24.97 (1.83; 5)	122.21 (6.63; 4)
17.5	5.9 (0.08; 29)	4.05 (0.14; 14)	4.34 (0.12; 14)	42.74 (4.51; 11)	19.11 (0.43; 10)	76.88 (4.99; 10)
20	4.29 (0.02; 40)	2.65 (0.04; 20)	3.12 (0.07; 19)	18.4 (0.5; 19)	15.51 (0.21; 17)	43.91 (0.57; 17)
22.5	3.33 (0.03; 40)	2.28 (0.07; 18)	2.46 (0.08; 18)	17.74 (0.62; 16)	12.25 (0.19; 13)	37.63 (0.58; 13)
25	2.82 (0.02; 37)	1.86 (0.04; 20)	2.32 (0.04; 20)	13.8 (0.38; 20)	9.57 (0.17; 18)	29.91 (0.74; 18)
27.5	2.58 (0.02; 40)	1.62 (0.04; 19)	2.08 (0.05; 19)	13.66 (0.49; 17)	8.07 (0.25; 13)	27.64 (0.48; 13)
30	2.18 (0.02; 36)	1.38 (0.02; 17)	1.66 (0.08; 17)	10.56 (0.47; 16)	7.48 (0.16; 8)	22.18 (0.42; 7)
32.5	2.1 (0.02; 35)	1.33 (0.03; 17)	1.78 (0.04; 17)	9.02 (0.58; 6)	-	-

Larval size, pupal weight, and adult size differed significantly between temperatures (larval length *F*_9.164_ = 13.267, *P* < 0.001, larval weight *F*_9.164_ = 26.944, *P* < 0.001, pupal weight *F*_7.95_ = 22.188, *P* < 0.001, adult length *F*_6.81_ = 13.243, *P* < 0.001, adult weight *F*_6.81_ = 11.777, *P* < 0.001; Table 2). The largest size of pupae and adult beetles was recorded at 17.5 °C, and then, beetle size decreased with temperature (Table 2). Larval size changed similarly; however, the largest larvae were reared at 15 °C (Table 2).

**Table 2:** Length and weight (mean (SE; *N*)) of *C. maxillosus* bred at different temperatures. Means were calculated based on the length and weight measurements conducted at the beginning of particular stage

Temperature (°C)	3rd-instar length	3rd-instar weight	Pupal weight	Adult length at emergence	Adult weight at emergence
10	16.85 (0.21; 10)	45.91 (1.97; 10)	–	–	–
12.5	18.29 (0.32; 12)	56.67 (2.79; 12)	–	–	–
15	20.53 (0.36; 20)	74.83 (2.82; 20)	177.9 (3.69; 12)	19.67 (0.58; 9)	147.69 (4.29; 9)
17.5	20.28 (0.29; 16)	69.72 (2.68; 16)	185.01 (6.63; 11)	21.1 (0.39; 10)	158.83 (6.01; 10)
20	19.88 (0.26; 20)	65.14 (1.48; 20)	160.86 (4.47; 16)	20.9 (0.34; 15)	140.73 (4.14; 15)
22.5	19.78 (0.29; 20)	62.88 (2.65; 20)	148.16 (4.83; 19)	19.65 (0.35; 17)	127.72 (3.95; 17)
25	19.39 (0.12; 19)	60.06 (1.19; 19)	146.7 (3.06; 19)	18.11 (0.26; 18)	124.65 (2.6; 18)
27.5	18.6 (0.29; 20)	51.07 (1.83; 20)	130.45 (5.25; 15)	17.61 (0.45; 14)	114.57 (8.04; 14)
30	19.13 (0.44; 19)	48.87 (1.88; 19)	110.16 (5.23; 10)	17.5 (0.59; 5)	94.56 (2.48; 5)
32.5	17.5 (0.25; 18)	38.18 (2.05; 18)	-	-	-

### Differences in the development of *C. maxillosus* fed with different types of food

**Fig. 3:**
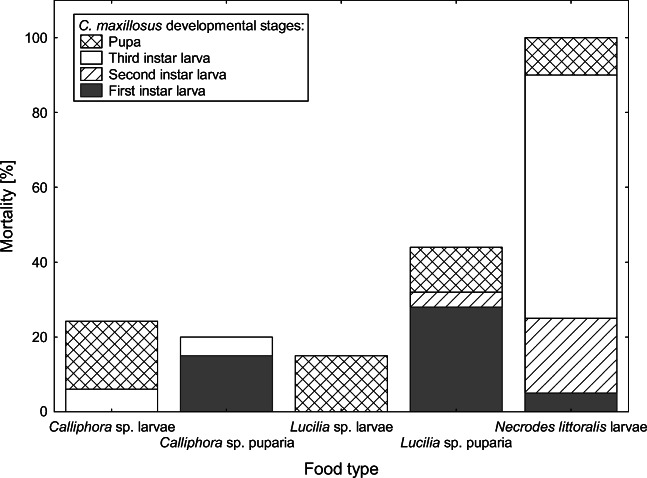
Mortality of *C. maxillosus* developmental stages reared at 24 °C and fed with different food types

Mortality significantly varied between types of food (*χ*^2^ = 131.4858, *P* < 0.001; Fig. 3). No beetles reached the adult stage when fed with larvae of *N. littoralis* (Fig. 3). The lowest mortality was observed for beetles fed with *Calliphora* sp. and *Lucilia* sp. larvae (Fig. 3). In the case of beetles fed with blowfly puparia, the highest mortality was observed for first-instar larvae (Fig. 3). In the case of beetles fed with blowfly larvae, the highest mortality was observed at the pupal stage (Fig. 3).

**Fig. 4:**
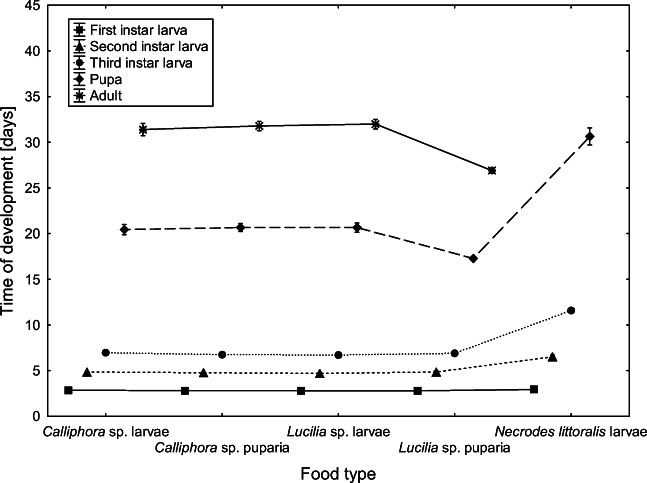
Duration of *C. maxillosus* developmental stages reared at 24 °C and fed with different food types. Vertical bars represent standard errors; symbols represent mean developmental times of different instars

First- and second-instar larvae of *C. maxillosus* fed with *N. littoralis* larvae developed significantly longer than beetles fed with other types of food (first instar *F*_4.89_ = 87.38, *P* < 0.001; second instar *F*_4.86_ = 228.79, *P* < 0.001; Fig. 4). Total development time was the shortest when *C. maxillosus* were fed with *Lucilia* sp. puparia (*F*_3.68_ = 13.166, *P* < 0.001; Fig. 4).

Length and weight of third-instar larvae were the lowest when they were fed with *Necrodes littoralis* larvae (length *F*_4.85_ = 59.456, *P* < 0.001, weight *F*_4.85_ = 44.607, *P* < 0.001; Table 3). Pupal and adult length and weight were the highest when larvae were fed with *Calliphora* sp. puparia; the differences between beetles fed with different blowfly-related food types were however insignificant (pupal weight *F*_3.69_ = 1.0929, *P* = 0.35805; adult length *F*_3.63_ = 0.30579, *P* = 0.82110; adult weight *F*_*3*.63_ = 1.2683, *P* = 0.29293; Table 3).

**Table 3:** Length and weight (mean (SE; *N*)) of *C. maxillosus* reared at 24 °C fed with different types of food. Means were calculated based on the length and weight measurements conducted at the beginning of particular stage

Food type	3rd-instar length	3rd-instar weight	Pupal weight	Adult length at emergence	Adult weight at emergence
*Calliphora* sp. larvae	20.08 (0.2; 24)	65.75 (2.38; 24)	174.72 (4.28; 23)	20.38 (0.28; 21)	149.52 (3.61; 21)
*Calliphora* sp. puparia	20.68 (0.26; 17)	71.17 (3.9; 17)	184.86 (9.12; 16)	20.66 (0.37; 16)	160.1 (8.84; 16)
*Lucilia* sp. larvae	20.6 (0.21; 20)	69.63 (1.96; 20)	176.24 (4.08; 20)	20.35 (0.28; 17)	149.01 (3.84; 17)
*Lucilia* sp. puparia	19.29 (0.23; 14)	59.71 (1.76; 14)	169.67 (4.25; 14)	20.65 (0.25; 13)	145.11 (4.42; 13)
*Necrodes littoralis* larvae	16.07 (0.18; 15)	28.19 (0.97; 15)	58.6 (15.75; 2)	-	-

### Development models

**Fig. 5:**
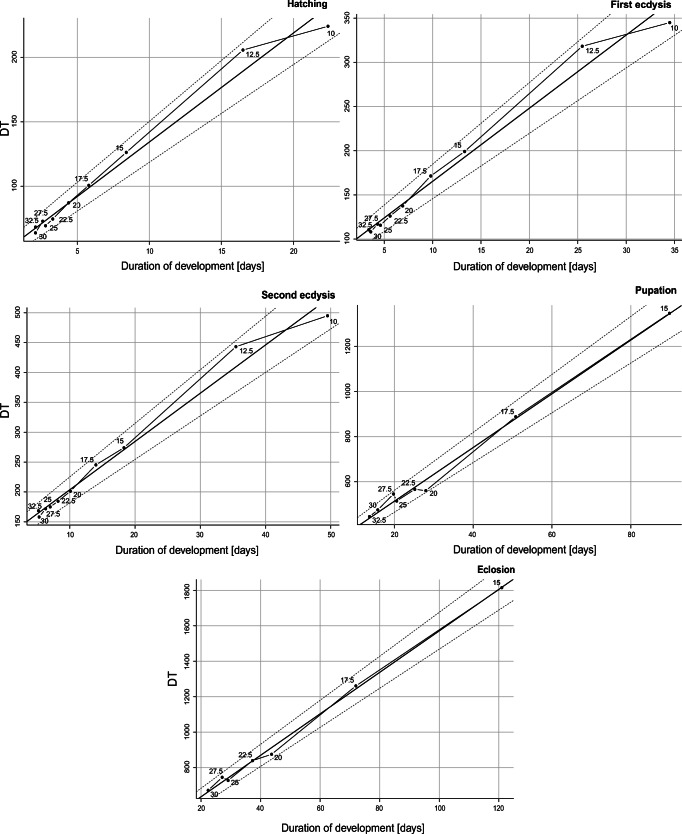
Reduced major axis (RMA) regression lines sensu Ikemoto and Takai [10] with 95% confidence intervals were used to determine thermal constants for five developmental events; DT is the time in days to reach the adult stage multiplied by the constant rearing temperature

All temperature points were included while calculating linear model parameters for developmental events (Fig. 5). Lower developmental thresholds calculated from linear model ranged from 8.085 ± 0.365 °C for the second ecdysis to 11.98 ± 0.351 °C for the pupation (Table 4). Although lower development threshold calculated from linear model was slightly below 12 °C, beetles failed to reach the adult stage already at 12.5 °C. Estimated thermal summation constants required to reach certain developmental events are presented in Table 4.

**Table 4:** Thermal summation models for five developmental events of *C. maxillosus* calculated using Ikemoto and Takai [10] method

	Model	Temp. range	*N*	Thermal summation constant *K* (SE)	Developmental threshold *D*_0_ (SE)	*r*^2^
Hatching	DT = 49.225 + 8.506 × D	10–32.5	10	49.225 (4.009)	8.506 (0.415)	0.981
First ecdysis	DT = 81.688 + 8.314 × D	10–32.5	10	81.688 (6.033)	8.314 (0.402)	0.981
Second ecdysis	DT = 122.883 + 8.085 × D	10–32.5	10	122.883 (7.80)	8.085 (0.365)	0.984
Pupation	DT = 274.825 + 11.984 × D	15–32.5	8	274.825 (14.49)	11.984 (0.351)	0.995
Eclosion	DT = 405.156 + 11.660 × D	15–30	7	405.156 (14.63)	11.660 (0.243)	0.998

**Fig. 6:**
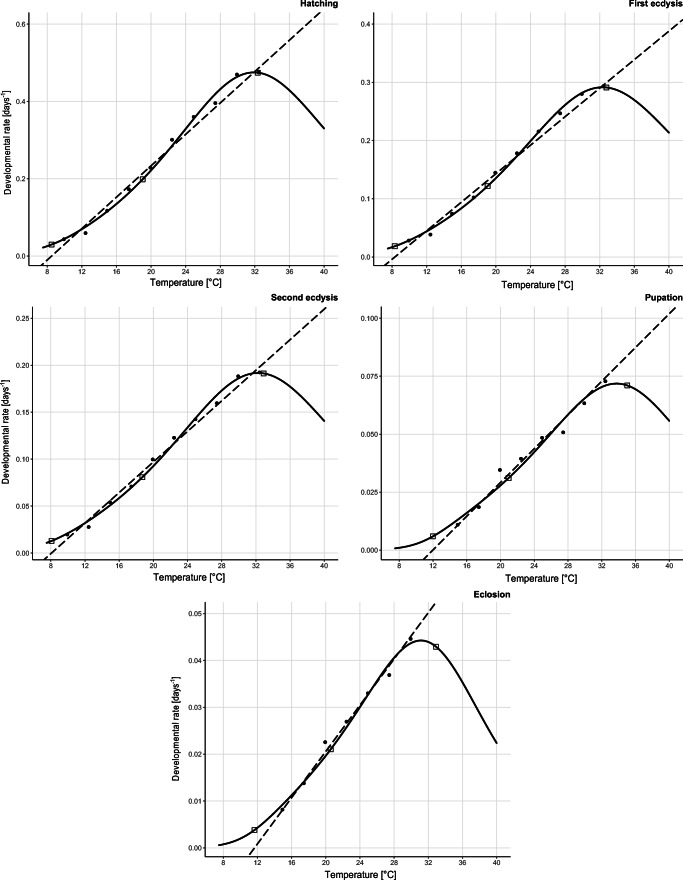
SSI models for five developmental events of *C. maxillosus*. Black circles represent observed developmental rates at particular rearing temperatures; open squares denote the predicted development rates at temperature at which the enzyme is 1/2 active and 1/2 low temperature inactive (°C) (*T*_L_), intrinsic optimum temperature at which no enzyme inactivation is hypothesized (°C) (*T*_φ_), and temperature at which the enzyme is 1/2 active and 1/2 high temperature inactive (°C) (*T*_H_)

In general, upper developmental thresholds were higher and lower developmental thresholds were lower when estimated with the Analytis, Brière-2, and Lactin-2 nonlinear models (Online Resource 1 and 2). Moreover, for pupation and eclosion, parameters of nonlinear models showed unrealistically high values of *T*_max_, reaching up to 185.8 °C (Online Resource 1). The SSI model provided more reliable parameter values, and the estimated intrinsic optimum temperature, *T*_φ_, ranged from 18.71 to 20.96 °C. Lower developmental thresholds *T*_min_ calculated from linear model and *T*_L_ calculated from the SSI model were highly congruent (Tables 4 and 5). Values of *T*_H_ calculated using the SSI model ranged from 32.35 °C for hatching to 35.03 °C for pupation (Table 5, Fig. 6).

**Table 5:** Estimated parameters and goodness of fit (AICc) of the SSI model for five developmental events of *C. maxillosus*

Parameter	Hatching	First ecdysis	Second ecdysis	Pupation	Eclosion
*ρ*_φ_	0.213748	0.1312873	0.08648179	0.032643	0.022
*ΔH*_A_	19260.18	18671.95	18005.09	18217.24	19132.11
*ΔH*_L_	− 52357.1	− 51441.47	− 53625.17	− 77343.7	− 73724.8
*ΔH*_H_	41899.41	40602.36	40045.81	44281.39	52283.35
*T*_φ_	19.0278	19.0387	18.7123	20.9551	20.5736
*T*_L_	8.506	8.3141	8.0852	11.9839	11.6601
*T*_H_	32.3455	32.7719	32.9047	35.0283	32.906
*AICc*	− 9.85	− 19.78	− 27.9	− 34.9725	− 35.48
*r*^*2*^	0.9946	0.99687	0.9936287	0.965429	0.981282

*ρ*_φ_*—*mean development rate at the intrinsic optimum temperature (1/day)

*ΔH*_A_—enthalpy of activation of the reaction that is catalyzed by the enzyme (cal/mol)

*ΔH*_L_—change in enthalpy associated with low temperature inactivation of the enzyme (cal/mol)

*ΔH*_H_—change in enthalpy associated with high temperature inactivation of the enzyme (cal/mol)

*T*_φ_—intrinsic optimum temperature at which no enzyme inactivation is hypothesized (°C)

*T*_L_—temperature at which the enzyme is 1/2 active and 1/2 low temperature inactive (°C)

*T*_H_—temperature at which the enzyme is 1/2 active and 1/2 high temperature inactive (°C)

### Validation

**Fig. 7:**
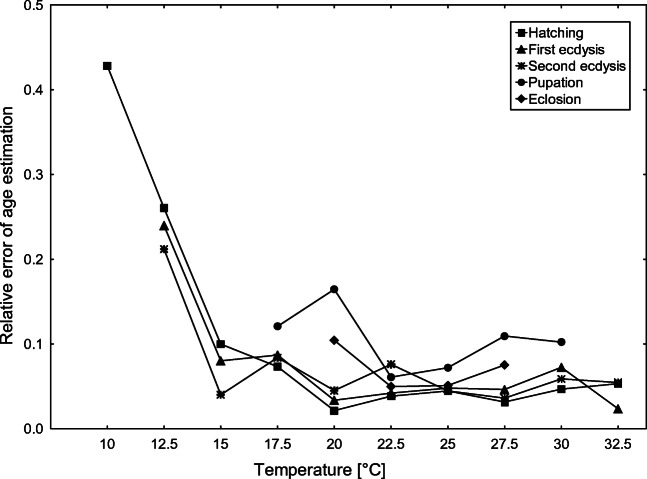
Relative error of age estimation for *C. maxillosus* validation specimens (reared at ten constant temperatures) using thermal summation data from our Table 4

The highest errors were recorded for beetles bred at low temperatures, i.e., for all developmental event errors were between 21 and 43% (Fig. 7). Starting from 15 °C, mean error rates were usually below 10% (Fig. 7). Mean error rates were higher for pupation and eclosion than those for hatching and first and second ecdyses (*F*_4.25_ = 16.421, *P* = 0.00000; Fig. 7).

**Fig. 8:**
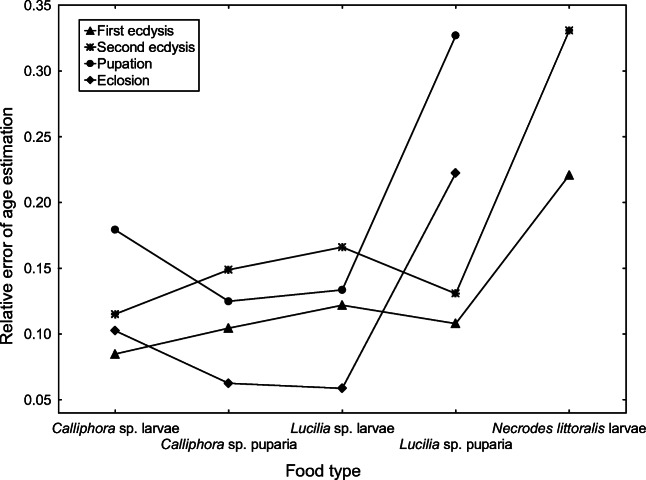
Relative error of age estimation for *C. maxillosus* validation specimens reared at 24 °C and fed with different food types. Thermal summation data from Table 4 were used for the estimation

The highest errors were recorded for beetles fed with *N. littoralis* larvae (22% for the first ecdysis and 33% for the second ecdysis) (Fig. 8). High error rates were also recorded for beetles fed with *Lucilia* sp. puparia (32% for pupation and 22% for eclosion) (Fig. 8).

## Discussion

We found that immature stages of *C. maxillosus* reached the adult stage in seven out of ten rearing temperatures (15–30 °C). The thermal requirements for *C. maxillosus* development have previously been investigated for populations from China [45] and Southern United States [44]. Wang et al. [45] investigated the development at the temperature range 17.5–32.5 °C and *C. maxillosus* reached the adult stage at all temperatures. Development times from oviposition to adult emergence for *C. maxillosus* in the present study were similar to these reported by Wang et al. [45]. The differences were found only at 17.5 °C and 20 °C. Total development time at 16 °C from Watson-Horzelski’s study [44] was half shorter (65.47 days) than in the present study (122.21 days at 15 °C). It is possible that these large differences are a result of the shift in the linear portion of the relation between temperature and rate of development. Perhaps in the case of the *C. maxillosus* population from the USA, 16 °C lies within the liner portion of the relationship, whereas in the case of the European population of *C. maxillosus*, 15 °C lies beyond this portion. Consequently, at the same low temperatures, Central European beetles should develop longer than beetles from the USA. In the current study, no specimen reached the adult stage when bred at 32.5 °C. At the same temperature, Wang et al. [45] and Watson-Horzelski [44] obtained adult specimens. It is probably due to the fact that these populations have wider ranges of acceptable temperatures. The differences in development of *C. maxillosus* between the studies could be also due to a number of other factors, e.g., differences in beetle diets or differences in rearing conditions. It is difficult to compare such research because of the large differences of the methods used. However, such comparisons reveal that standardization of the insect rearing protocols seems to be one of the most pressing needs in forensic entomology.

Size of pupae and emerging adult beetles was inversely related to temperature. Starting from 17.5 °C, pupae and adult beetles became smaller. Similarly, larval size decreased with temperature starting from 15 °C. These findings are consistent with the temperature-size rule (“hotter is smaller”) and represent a form of phenotypic plasticity commonly occurring in ectotherms [19].

As expected, the food type influenced the development of *C. maxillosus*. First- and second-instar larvae of the beetles fed with *N. littoralis* larvae developed significantly longer than larvae fed with other diets. Additionally, no specimen reached the adult stage when fed with *Necrodes littoralis* larvae. These results indicate that in natural and typical conditions, *C. maxillosus* larvae do not prey on larval *Necrodes littoralis*. When larvae were fed with blowfly larvae, the highest mortality was observed at the beetle pupal stage (Fig. 3). However, for *C. maxillosus* larvae fed with blowfly puparia, the highest mortality was observed at the first larval stage of *C. maxillosus* (Fig. 3). Probably, only some of the first-instar larvae were able to puncture the blowfly puparium, and for this reason, beetle mortality at this stage was so high. These findings indicate that *C. maxillosus* may change food preferences during its development. First-instar larvae may feed on blowfly larvae only, while second- and third-instar larvae may switch to a more diverse diet, with larger contribution of blowfly puparia. These patterns are actually consistent with successional patterns of blowflies and *C. maxillosus* as recorded on pig carcasses [57].

Current linear models are useful for minimum PMI estimation in Central Europe. The thermal constant *K* for the total development time was lower than that in Wang et al.’s study [45] (405.16 ± 14.63 and 492.06 ± 23.61 ° days, respectively). Lower developmental threshold (*T*_min_) was however higher than the one presented by Wang et al. [45] (11.6 ± 0.24 °C and 9.6 ± 0.58 °C, respectively). Due to the lower *T*_min_ for *C. maxillosus* from China, it should logically take longer to reach the adult stage. These differences may therefore result from differences in thermal requirements of the Chinese and Central European populations of the beetle. However, lower developmental threshold for the Chinese population of *C. maxillosus* might have been underestimated due to the poor representation of the low temperatures in this study (i.e., 17.5–32.5 °C).

In our study, *T*_min_ and *T*_max_ for pupation and eclosion obtained from nonlinear models (Analytis, Brière-2, and Lactin-2) were not satisfactory, since some of them represented biologically unrealistic values (see Online Resource 1), e.g., very low values for *T*_min_, some even below 0 °C (see Online Resource 1). Our fit of nonlinear models was biased in the region close to upper development rate limits. On the other hand, in the similar study on *Fannia canicularis* (Linnaeus) (Diptera: Fanniidae) [17], nonlinear models were biased in the area of lower development rate limits. Because of the high mortality of *C. maxillosus* in the highest examined temperatures, data used for nonlinear modeling represented a rather straight line and, in consequence, led to a failure in nonlinear model fitting. Estimating lower and upper thermal thresholds with the use of empirical nonlinear models, e.g., the Analytis, Brière-2, or Lactin-2, may not provide reliable results [59]. Application of nonlinear models to determine thermal tolerance of forensically useful insects, i.e., the temperature range between the minimum and the maximum rate of development is therefore not a useful approach. Simpler linear models provide more reliable estimations of *T*_min_ and additionally allow estimating *K*, a constant of primary usefulness for forensic practice. On the other hand, the theoretical SSI model may provide forensically useful information on the development of examined insect species [59].

Environmental conditions used in reference developmental studies are rarely the same as those experienced during casework. Thus, accuracy of lab-generated data should be determined preferably in a robust validation study. Validation part of this study revealed that accuracy in age estimation was lower using models for final development landmarks, i.e., pupation or eclosion than models for earlier events. Interestingly, age estimation errors were the largest while using model for pupation (Fig. 7). This may be due to the large variation in duration of the third larval stage resulting from surprisingly high variation in the postfeeding larval phase (Table 1).

Because the food type influenced the development of *C. maxillosus* similarly, it must have affected the accuracy of age estimation for specimens reared on different diets. Developmental models were created based on specimens fed with *Calliphora* sp. larvae. When models were validated using specimens fed with the same type of food and in the same laboratory trials, error rates were below 10% for all developmental events (see data for 22.5 and 25 °C in Fig. 7). However, when we used specimens fed with different diets and reared under 24 °C in different trials, error rates were generally larger (usually between 10 and 15%), and for some diets, they were very much larger, e.g., 32% for pupation and 22% for eclosion for larvae fed with *Lucilia* sp. puparia (Fig. 8). As for the fly-related diets, total development times were the shortest (Table 3), mortality was the highest (Fig. 3), beetle size was the smallest (Table 4), and estimation errors were the largest (Fig. 8) for *C. maxillosus* fed with *Lucilia* puparia. These findings indicate that in natural and typical conditions, larvae of *C. maxillosus* do not prey solely on *Lucilia* puparia, probably due to the low quality of this diet. Some studies suggest that food quality affects thermal constants in insects [60–62]. Additionally, Jarošík et al. [60] showed that aphidophagous ladybirds develop significantly faster and start developing at significantly lower temperature on a good-quality diet. Consequently, the food quality may affect lower development threshold, indicating that the threshold may change depending on a diet. Similar results were reported for moths [61, 62] and aphids [63]. It is therefore possible that similar effects may occur in case of *C. maxillosus* and other forensically useful insects. In natural conditions, carrion insects usually have access to optimal diet. Therefore, insect evidences encountered in casework are usually specimens fed with optimal food type. However, the problem may arise when we estimate their age with developmental data obtained using non-optimal diet. This source of estimation error may be particularly important in case of predatory insects, as was demonstrated in this article for predatory *C. maxillosus* beetle. Moreover, these findings indicate that collecting reference developmental data using optimal diet is crucial and that forensic entomologists should pay more attention to the quality of food used in developmental studies and in rearing insects during casework.

## Electronic supplementary material

ESM 1 (PDF 245 kb)

ESM 2 (PDF 494 kb)
