# Supplementary material for: Development and validation of forensically useful growth models for Central European population of Creophilus maxillosus L. (Coleoptera: Staphylinidae)
Source: Int J Legal Med. 2020 Apr 8;134(4):1531–45. doi: 10.1007/s00414-020-02275-3 (PMC7295842; doi:10.1007/s00414-020-02275-3)
Supplement: Supplementary file 1 — (PDF 245 kb) [file 414_2020_2275_MOESM1_ESM.pdf]

SUPPLEMENTARY TABLES FOR

Development and validation of forensically useful growth models for Central European population of *Creophilus maxillosus* L. (Coleoptera: Staphylinidae)

Katarzyna Frątczak-Łagiewska[1-3*], Andrzej Grzywacz[4], Szymon Matuszewski[1-2]

[1]Laboratory of Criminalistics, Adam Mickiewicz University, Św. Marcin 90, 61-809 Poznań, Poland

[2]Centre for Advanced Technologies, Adam Mickiewicz University, Uniwersytetu Poznańskiego 10, 61-614 Poznań, Poland

[3]Department of Animal Taxonomy and Ecology, Adam Mickiewicz University, Uniwersytetu Poznańskiego 6, 61-614 Poznań, Poland

[4]Department of Ecology and Biogeography, Faculty of Biological and Veterinary Sciences, Nicolaus Copernicus University, Lwowska 1, 87-100 Toruń, Poland

*Corresponding author: katarzyna.fratczak@amu.edu.pl

Content

Table 1. Estimated parameters and goodness of fit (*AICc*) of the nonlinear developmental models for five developmental events of *C. maxillosus*.

Model	Parameter	Hatching	First ecdysis	Second ecdysis	Pupation	Eclosion
Analytis	*a*	0.0006601	0.00001843	0.00003636	$2.665*10^{-24}$	$2.75*10^{-33}$
	n	0.5332	0.4214	0.4868	0.1004	0.1003
	m	3.721	1.458	1.493	0.1709	0.1155
	T_{min}	2.7	2.46	3.63	-2.027	-16.73
	T_{max}	42.27	42.89	44.71	48.43	77.99
	T_{fast}	33.17	33.83	34.60763	29.75	33.98
	AICc	-85.19	-104.52	-102.91	-65.05	-82.08
Brière-2	*a*	0.003927	0.000210755	0.00008685	$4.994*10^{-18}$	$1.185*10^{-7}$
	d	3.638	3.30341	2.227	0.1643	0.5709
	T_{min}	6.298	5.8819	5.663	10.78	10.64
	T_{max}	36.16	37.36	40.62	185.8	69.73
	T_{fast}	32.23	32.89	33.75	50.45	40.04
	AICc	-87.03	-105.57	-104.85	-86.05	-87.6
Lactin-2	*P*	0.1206	0.11875	0.10366	0.01043	0.008049
	ΔT	8.1806	8.35294	9.56126	52.63348	12.35552
	λ	-0.1166	-0.06909	-0.06475	-0.71485	-0.03712
	T_{max}	40.15	40.65775	42.34	129.31	46.84
	T_{fast}	31.85	32.21	32.67	35	34.37
	AICc	-85.08	-101.79	-103.62	-87.06	-86.81

T_{min} is the lower developmental threshold, T_{max} is the upper developmental threshold, T_{fast} is the temperature of the fastest development the and *a*, *n*, *m*, *d*, *p*, ΔT and λ are estimated model parameters.
