# Supplementary material for: Development and validation of forensically useful growth models for Central European population of Creophilus maxillosus L. (Coleoptera: Staphylinidae)
Source: Int J Legal Med. 2020 Apr 8;134(4):1531–45. doi: 10.1007/s00414-020-02275-3 (PMC7295842; doi:10.1007/s00414-020-02275-3)
Supplement: Supplementary file 2 — (PDF 494 kb) [file 414_2020_2275_MOESM2_ESM.pdf]

SUPPLEMENTARY FIGURES FOR

Development and validation of forensically useful growth models for Central European population of *Creophilus maxillosus* L. (Coleoptera: Staphylinidae)

Katarzyna Frątczak-Łagiewska[1-3*], Andrzej Grzywacz[4], Szymon Matuszewski[1-2]

[1]Laboratory of Criminalistics, Adam Mickiewicz University, Św. Marcin 90, 61-809 Poznań, Poland

[2]Centre for Advanced Technologies, Adam Mickiewicz University, Uniwersytetu Poznańskiego 10, 61-614 Poznań, Poland

[3]Department of Animal Taxonomy and Ecology, Adam Mickiewicz University, Uniwersytetu Poznańskiego 6, 61-614 Poznań, Poland

[4]Department of Ecology and Biogeography, Faculty of Biological and Veterinary Sciences, Nicolaus Copernicus University, Lwowska 1, 87-100 Toruń, Poland

*Corresponding author: katarzyna.fratczak@amu.edu.pl

Content

Fig. 1. Lactin-2 models fit to observed developmental rates of five development events of *C. maxillosus*. Black circles indicate observed data.

Fig. 2. Brière-2 models fit to observed developmental rates of five development events of *C. maxillosus*. Black circles indicate observed data.

Fig. 3. Analytis models fit to observed developmental rates of five development events of *C. maxillosus*. Black circles indicate observed data.

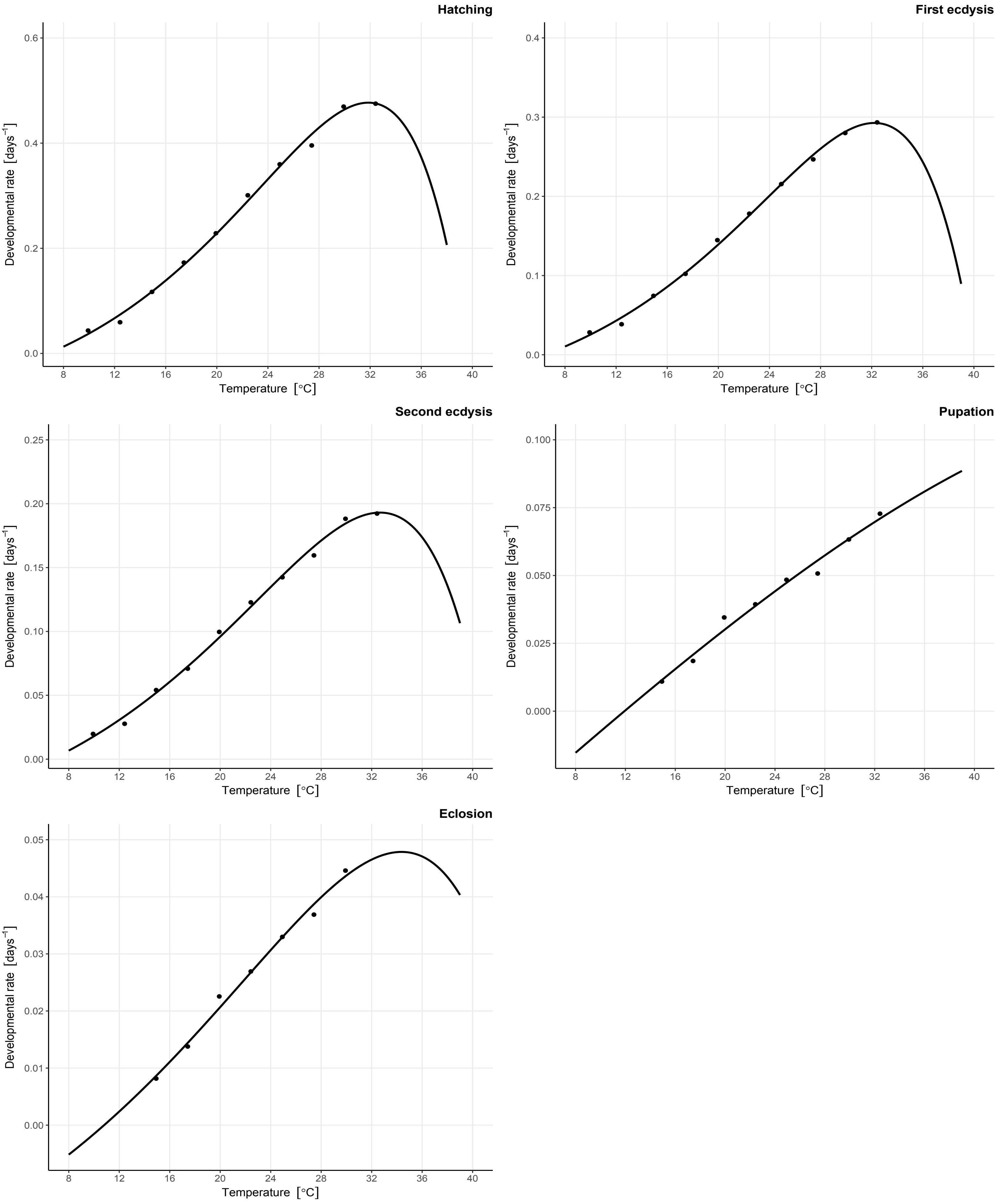

Fig. 1. Lactin-2 models fit to observed developmental rates of five development events of *C. maxillosus*. Black circles indicate observed data.

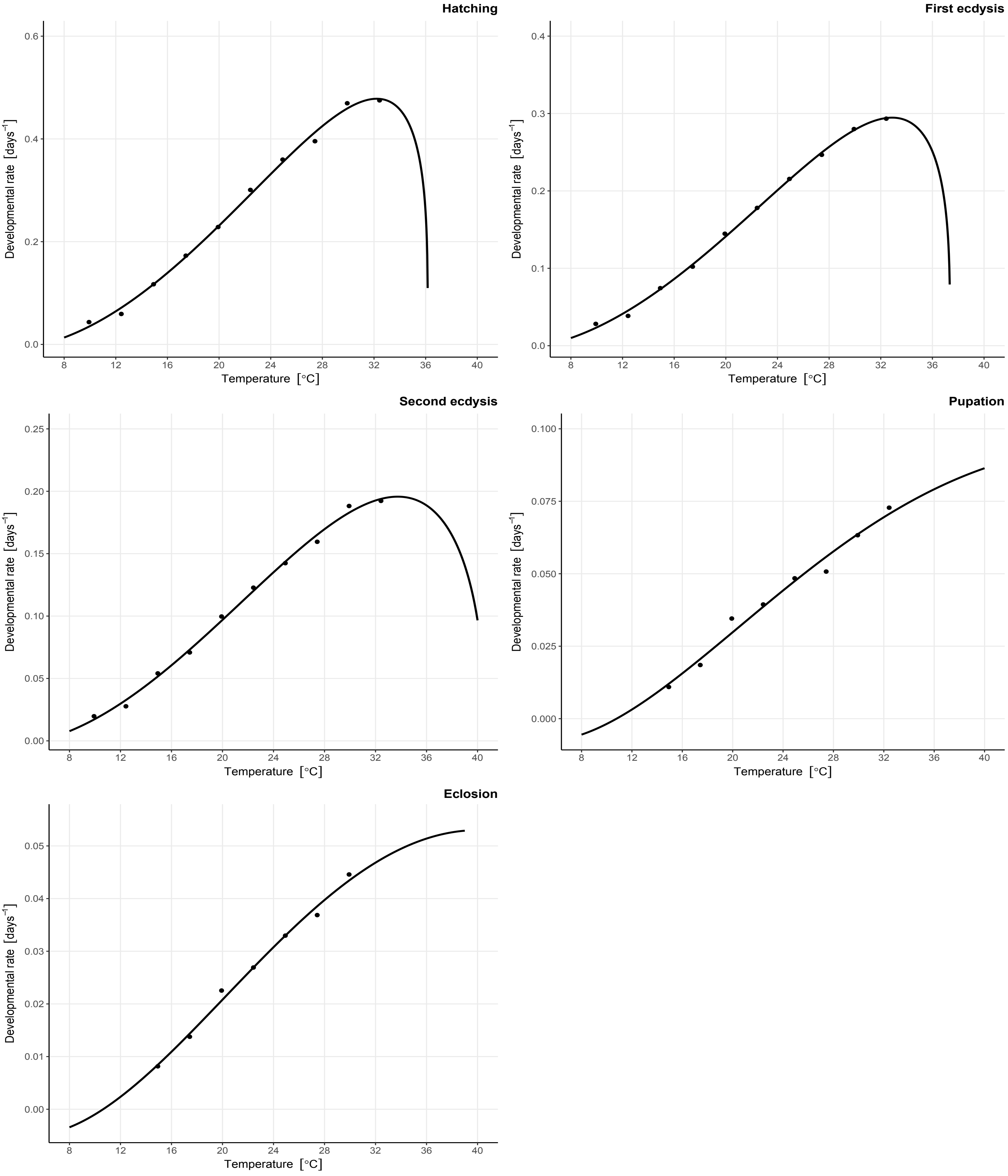

Fig. 2. Brière-2 models fit to observed developmental rates of five development events of *C. maxillosus*. Black circles indicate observed data.

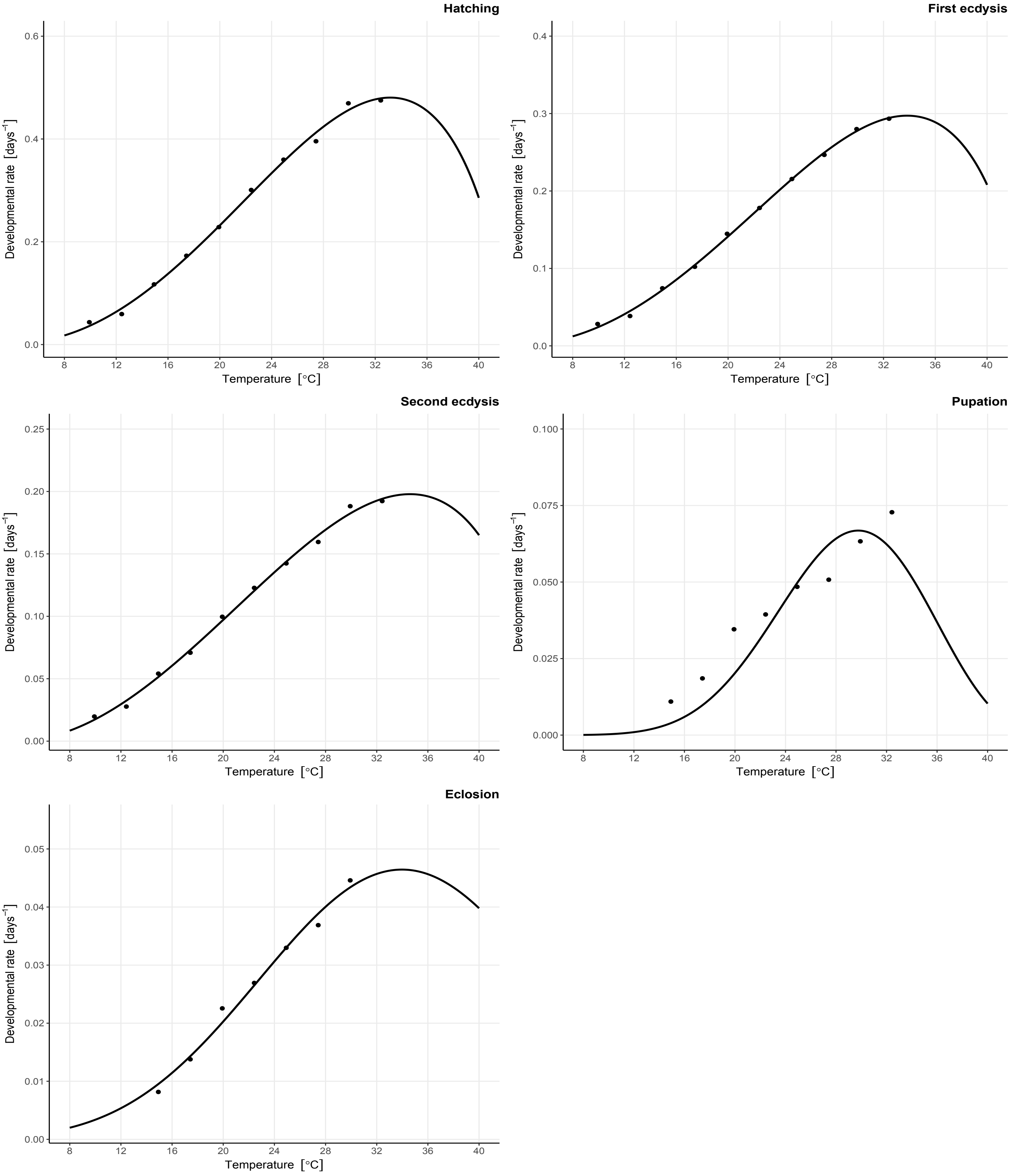

Fig. 3. Analytis models fit to observed developmental rates of five development events of *C. maxillosus.* Black circles indicate observed data.
